# Temporomandibular Disorder Prevalence and Its Association with Lifestyle Habits in Biomedicine Students—A Cross-Sectional Study

**DOI:** 10.3390/healthcare11162261

**Published:** 2023-08-11

**Authors:** Ivan Frka Separovic, Dinko Martinovic, Antonella Lesin, Ema Puizina Mladinic, Daria Tokic, Marko Kumric, Laura Jurina, Marino Lupi-Ferandin, Josipa Bukic, Josko Bozic

**Affiliations:** 1 Study of Dental Medicine, University of Split School of Medicine, Soltanska 2, 21000 Split, Croatia; 2 Department of Maxillofacial Surgery, University Hospital of Split, 21000 Split, Croatia; 3 Department of Anesthesiology and Intensive Care, University Hospital of Split, 21000 Split, Croatia; 4 Department of Pathophysiology, University of Split School of Medicine, 21000 Split, Croatia; 5 Department of Pharmacy, University of Split School of Medicine, 21000 Split, Croatia

**Keywords:** temporomandibular disorder, biomedicine students, lifestyle habits, Fonseca anamnestic index, jaw functional limitation scale, perceived stress questionnaire

## Abstract

This study aimed to examine the frequency of temporomandibular disorder among biomedical students and relate its occurrence to lifestyle habits. A cross-sectional collection of data was carried out and included a total of 676 examinees through a questionnaire that had 73 questions: general information and lifestyle habits, the Fonseca Anamnestic index (FAI), the Jaw Function Limitation Scale (JFLS), and the Perceived Stress Questionnaire (PSQ). The statistical analyses between three or more groups were conducted using the one-way analysis of variance (ANOVA) with post hoc Scheffé test or Kruskal–Wallis test with post hoc Dunn’s test for quantitative variables. The comparison of qualitative variables was conducted using the Chi-square test, while the correlations were determined using Spearman’s correlation test. The analysis showed that a higher frequency of moderate or severe TMD was observed in subjects who were smokers (*p* < 0.001) compared to non-smokers. Subjects who consumed more coffee had moderate to severe TMD compared to subjects who consumed a lesser amount (*p* < 0.001). Furthermore, a positive correlation between the amount of stress and the severity of TMD was found. Our study implies that students of biomedical studies have an increased risk for TMD and that there is a link with their lifestyle habits.

## 1. Introduction

Temporomandibular disorders (TMDs) are a term that encompasses a wide spectrum of symptoms that are triggered by abnormal conditions in the temporomandibular joint (TMJ), mastication muscles and all related structures [1,2]. There is high variability in TMD prevalence in scientific studies; however, most of them report that 40–60% of the general population has one or more TMD symptoms, while 25% are complaining of pain in the temporomandibular area [3]. Furthermore, TMD is regarded as the second most widespread musculoskeletal disorder that causes disability and pain with a major impact on the patient’s quality of life [4,5]. Its etiology is considered multifactorial and significantly related to genotype and phenotype; however, several risk factors predispose, precipitate, or prolong TMD [6]. These are biological factors (e.g., sex hormones), age, endogenous opioid function, anatomical genotype differences, trauma, occlusal changes, systemic illness, parafunctions and psychosocial factors (e.g., exposure to stress, coping with pain, disasters, and emotions) [7].

Symptoms and signs of TMD include limited and/or abnormal range of motion, clicking, popping, or crepitus in function with or without locking of the jaw, TMJ pain, jaw opening pain, orofacial pain, otalgia, tinnitus and headache [8]. Moreover, these symptoms may overlap with other chronic pain disorders such as fibromyalgia, headaches and neurological conditions [9,10]. TMD is usually diagnosed using clinical signs and symptoms, along with behavioral and psychosocial status [11,12]. Nowadays, the Diagnostic Criteria for Temporomandibular Disorders (DC/TMDs) is the most broadly used and standardized tool for the evaluation and classification of TMD [13]. DC/TMD categorizes TMD into three groups: group I includes muscle disorders (including myofascial pain with and without mouth opening limitations); group II includes disc displacement with or without reduction and mouth opening limitations; and group III incorporates arthralgia, arthritis, and arthrosis [14]. Treatment for TMD includes conservative treatments such as medications, physical therapy, acupuncture, cognitive behavioral therapy, patient education, self-management techniques, splints, and invasive treatments such as surgery [15,16,17,18,19,20,21].

Lifestyle habits and lifestyle choices have an undeniable impact on an individual’s health. Sometimes even the smallest change in them can contribute to the development of various disorders and diseases. In addition to the proven etiological factors for the development of TMD, certain lifestyle habits can increase or worsen the symptoms of TMD and have recently been a focus in research associated with chronic pain and TMD. A study by Lei J. et al. showed a moderate to strong cross-age relationship between stress, depression and anxiety and TMD. Also, in patients with painful TMD, a greater amount of stress and reduced quality of life have been reported precisely because of the symptoms of the disorder itself [22].

It is common knowledge that smoking is positively related to alcohol consumption and that both have a negative effect on an individual’s health. In addition, smoking and increased alcohol intake are correlated with an unhealthy lifestyle. There are just a few studies linking alcohol consumption and smoking to TMD [23]. Some of the studies found that smokers with TMD report higher pain severity than nonsmokers or that smoking could be associated with a higher risk for TMD in young adulthood [24]. According to a study by Miettinen et al., the prevalence of TMD symptoms is linked with an increased frequency of alcohol consumption, and alcohol consumption once a week or more is often significantly associated with TMD symptoms. In addition, smoking was shown to be significantly associated with all TMD symptoms except TMJ clicking [25]. The direct effect of caffeine on TMD is not sufficiently covered in the scientific literature and requires additional research in this area, although it is well known that caffeine ingestion has an ergogenic effect that is probably mediated through the binding of caffeine to adenosine receptors [26].

Hence, the primary aim of this study was to determine the prevalence of temporomandibular joint disorder among biomedical students and whether there is an association with lifestyle habits such as tobacco, alcohol, coffee and energy drink usage. Furthermore, we wanted to evaluate is there a connection with the stress that students perceive.

## 2. Materials and Methods

### 2.1. Ethical Consideration and Study Design

This cross-sectional survey-based study was conducted at the University of Split School of Medicine in July 2022. Students of all years of medicine, dental medicine, and pharmacy studies at the University of Split School of Medicine were included.

The Ethics Committee of the University of Split School of Medicine approved the protocol of this research (Class: 003-08/22-03/0003, Reg. no.: 2181-198-03-04-22-0045), and it was conducted according to the Declaration of Helsinki. Finally, submitting all responses in the Google Forms^®^ survey application was considered informed consent, which was emphasized to the participants.

The research inclusion criteria were students of biomedical studies in Split older than 18 years. The exclusion criteria were: previous TMD diagnosis by clinical examination; congenital and acquired malformations of the mastication apparatus.

This study included a total of 676 students in biomedical studies. The link for the online Google Forms^®^ survey was distributed using messages and by e-mail to representatives of all study years of the faculty’s constituents. Participation was voluntary and anonymous.

### 2.2. Survey Questionnaire

The questionnaire consisted of 73 questions, divided into four parts. The first part of the questionnaire obtained general information, such as gender, age, year and type of study program. Information was also collected on certain lifestyle habits regarding smoking and consumption of alcohol, coffee and energy drinks.

The second part was the Fonseca Anamnestic Index (FAI). It is a validated, reliable tool that evaluates the existence of TMD and its severity based on the signs and symptoms of the disorder [27]. It contains 10 items to which respondents answer with one of three possibilities: yes (10 points), sometimes (5 points), or no (0 points). The final result is obtained by adding up all answers and classifying them into one of four categories: no TMD (0–15 points), mild TMD (20–45 points), moderate TMD (50–65 points), and severe TMD (70–100 points). In addition, FAI use has been recommended for screening patients with TMD in public health because of its simplicity.

The third part was the Jaw Function Limitation Scale (JFLS-20) [28]. The JFLS-20 is a validated questionnaire that consists of twenty items and assesses limitations in jaw mobility, chewing and verbal and emotional expression. For each offered item, the respondents had to mark the degree of limitation in performing a certain action during the last month from 0 to 10.

The fourth and last part of the questionnaire was the Perceived Stress Questionnaire (PSQ) [29]. It is a validated and reliable tool that consists of thirty items about stressful situations, to which subjects answer about the frequency of them during the last month. This questionnaire is used to assess stressful events that could affect the onset or worsening of disease symptoms. Participants answer using a scale from 1 (“rarely”) to 4 (“usually”) about how often they experience certain feelings related to stress. The total score is calculated by summing all items (however, items 1, 7, 10, 13, 17, 21, 25 and 29 are scored in the opposite direction, from 4 points (“rarely”) to 1 point (“usually”)). The final PSQ index is calculated by deducting 30 from the total score and dividing that by 90, giving an index between 0 and 1. The higher score is indicative of higher stress.

### 2.3. Statistical and Sample Size Analyses

The appropriate sample size was estimated using the online Surveymonkey^®^ calculator. The population of biomedical students at the University of Split School of Medicine that was eligible for this study was 909. Using the 95% confidence interval and a 5% margin of error, it was estimated that the collected sample must consist of at least 271 biomedical students. Since we included 676 students, with an even larger sample size, we have further improved the power of this study.

Statistical analysis of the collected data was performed using the computer software MedCalc (MedCalc version 20.114, Ostend, Belgium). All qualitative variables are presented as whole numbers and percentages, while continuous quantitative variables are presented as arithmetic mean ± standard deviation and non-continuous as median (interquartile range). The Kolmogorov–Smirnov test was used to estimate the normality of the distribution of quantitative variables. The comparison of qualitative variables between groups was conducted using the Chi-square test. Comparison of continuous variables between three or more groups was performed using one-way analysis of variance (ANOVA) with a post hoc Scheffé test, while analysis of non-continuous variables was performed using the Kruskal–Wallis test with a post hoc Dunn’s test. The association between non-parametric variables was determined using Spearman’s correlation test. The level of statistical significance was set at a *p*-value < 0.05.

## 3. Results

This study included 351 medical students, 148 dental medicine students, and 177 pharmacy students. In comparison to sociodemographic data, medical students had the largest ratio of male students (25.1%), while pharmacy had the lowest (15.8%) (Table 1). Furthermore, most medical students included were from the 2nd year (25.9%), most dental medicine students were from the 6th year (20.4%), and most pharmacy students were from the 3rd year (31.1%) (Table 1).

Regarding habits, in comparison between the three studies, there was a statistically significant difference in the smoking period (*p* = 0.001) and the number of cigarettes per day (*p* = 0.026). Dental medicine students showed the longest period of smoking (10.2 ± 5.7 years), and there was a significant difference with the other two groups. Furthermore, dental medicine students also showed a significantly higher period of coffee consumption (6.8 ± 3.5) compared to the other two groups. There were no other significant differences regarding lifestyle habits between the studies (Table 1).

**Figure 1:**
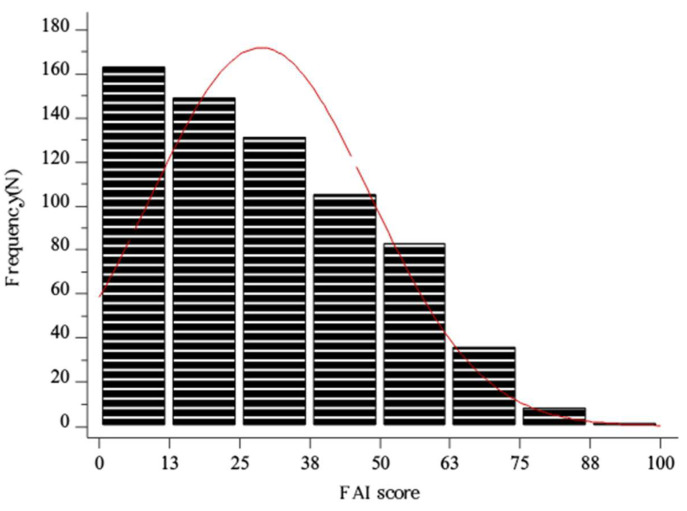
Histogram showing the distribution of FAI score in the whole study sample (N = 676). Red line–distribution.

**Figure 2:**
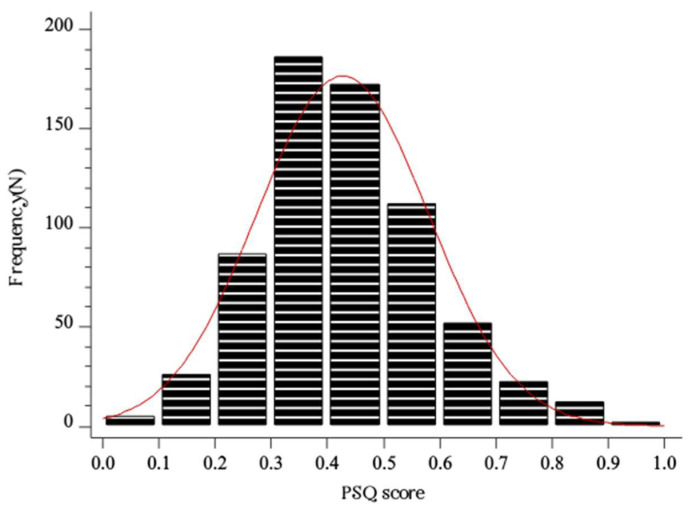
Histogram showing the distribution of PSQ score in the whole study sample (N = 676). Red line–distribution.

The mean FAI score in the study sample was 28.9 ± 19.6 (Figure 1). The median PSQ score in the study sample was 0.41 (0.32–0.57) (Figure 2).

The study sample was divided depending on the FAI score into four TMD severity groups (no TMD—FAI 0–15; minor TMD—FAI 20–45; medium TMD—FAI 45–65; and major TMD—FAI 70–100). Regarding lifestyle habits, there was a statistically significant difference (*p* = 0.014) in the ratio of smokers, as the major TMD group had the largest number (39.4%) (Table 2). Furthermore, both the major TMD group and the medium TMD group had the statistically longest smoking period, which was significantly higher (*p* < 0.001) compared to the other two groups (Table 2). Additionally, major TMD and medium TMD groups consumed the highest number of coffee cups per day, with a significant difference from the other two groups (Table 2). However, there were no significant differences regarding alcohol and energy drink consumption between the four groups (Table 2).

In the comparison of the JFLS score between the TMD severity groups depending on the FAI score, there was a significant difference between all groups in the total JFLS score (*p* < 0.001) (Table 3). Moreover, there was a significant difference regarding the all three subdimensions of the JFLS questionnaire (*p* < 0.001) (Table 3).

**Figure 3:**
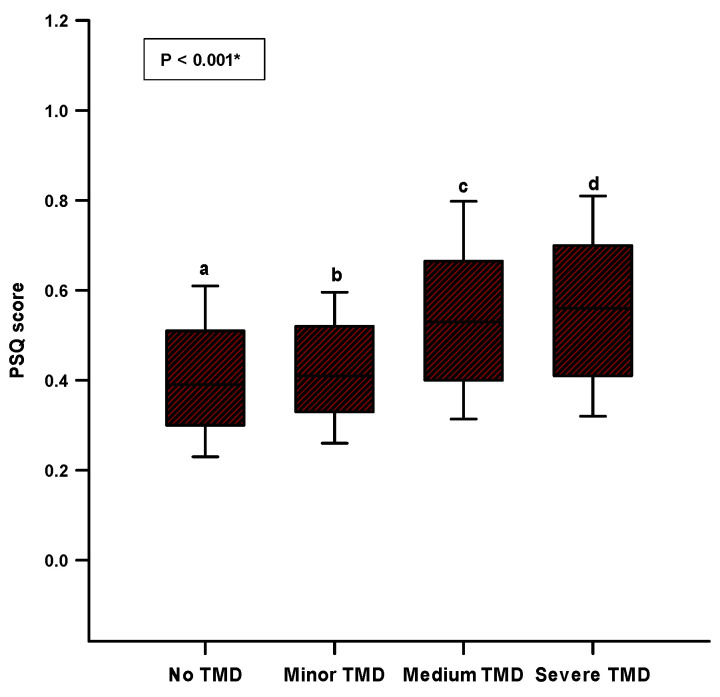
Comparison of PSQ scores between TMD severity groups depending on the FAI score. Abbreviations: TMD—temporomandibular disorder; PSQ—perceived stress questionnaire; FAI—Fonseca anamnestic index. * Kruskal–Wallis test with post hoc Dunn’s test. a vs. c/a vs. d/b vs. c/b vs. d = *p* < 0.05.

There was a significant difference (*p* < 0.001) in the comparison of the PSQ score between the groups of TMD severity depending on the FAI score (Figure 3). Medium and severe TMD had significantly higher PSQ scores, and a significant difference was found with the minor TMD and no TMD groups (no TMD—0.39 (0.30–0.51); minor TMD—0.41 (0.33–0.52); medium TMD—0.53 (0.40–0.66); and major TMD—0.56 (0.41–0.70)) (Figure 3).

**Figure 4:**
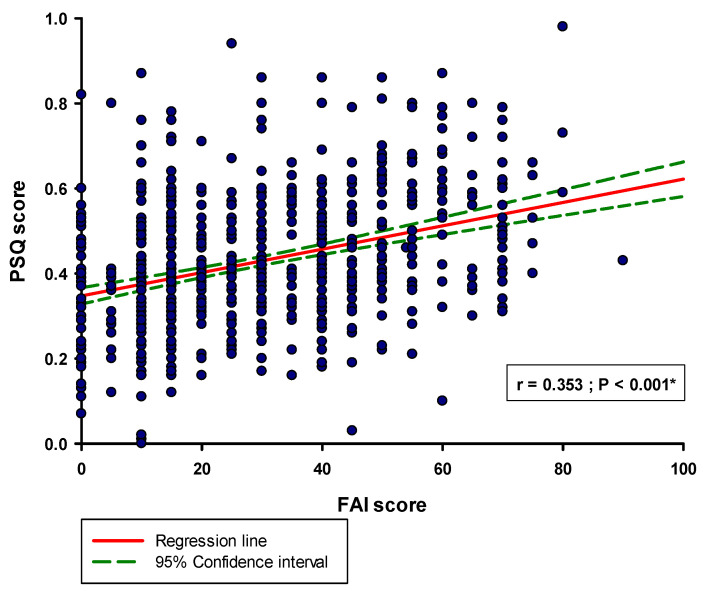
Correlation between PSQ score i FAI score (N = 676). Abbreviations: PSQ—perceived stress questionnaire; FAI—Fonseca anamnestic index. * Spearman’s correlation.

Furthermore, there was a significant positive correlation between the PSQ score and the FAI score (r = 0.353; *p* < 0.001) (Figure 4).

Furthermore, multivariable logistic regression showed that smoking (OR 0.564, 95% CI 0.306–1.040, *p* = 0.046), coffee consumption (OR 0.548, 95% CI 0.310–0.969, *p* = 0.038), PSQ score (OR 0.062, 95% CI 0.014–0.282, *p* < 0.001), and JFLS score (OR 0.923, 95% CI 0.891–0.957, *p* < 0.001) were significant negative predictors of not having TMD when computed along with baseline characteristics (Table 4).

## 4. Discussion

This research indicates the connection between lifestyle habits and TMD in the population of students of medicine, dental medicine, and pharmacy at the Faculty of Medicine, University of Split. Namely, students who declared themselves smokers more often had moderate or severe TMD categories compared to nonsmokers. However, there was no difference in the number of cigarettes smoked per day. The most important factor was the length of the smoking experience, so those who were long-term smokers had more severe forms of TMD.

Furthermore, in the case of coffee consumption, there was no association between the occurrence of TMD and whether coffee was consumed or not, or even for how long. However, more serious forms of TMD are often observed in students who consume more cups of coffee daily. So those who drink more coffee have a higher frequency of severe and medium forms of TMD.

It is also interesting that severe TMD was more common in medical and dental students compared to pharmacy students. In general, most students rarely use energy drinks. Alcohol consumption, although not generally recommended for patients with TMD, according to the results of this study, was not a predictor of the occurrence of TMD or of more severe forms of the disorder itself in affected students.

Previous research on the relationship between TMD and lifestyle habits in the student population, as well as other adolescent populations, is still inconclusive regarding the relationship between the severity of these disorders and lifestyle habits such as smoking or consumption of alcohol and coffee [25,30,31]. Smoking was associated with more severe forms of TMD and higher levels of stress in medical students, according to a study by Benoliel et al. [31]. Moreover, smoking and an increased frequency of alcohol consumption were associated with TMD symptoms among young Finnish adults [25]. Future research on the influence of lifestyle habits on TMD could include other characteristics, such as physical activity, marital status, and the presence of other diseases associated with the frequency of TMD, such as rheumatoid arthritis.

The results of this study also showed an association between the severity of TMD and the perception of stress in a population of medical, dental, and pharmacy students. Similar results were found in a study by Medeiros and colleagues, where a high frequency of TMD and symptoms of anxiety and depression were observed in the student population [32]. However, the mentioned research was conducted at the beginning of the pandemic and during the quarantine; therefore, there is a possibility that the results found were influenced by new stressors (the newly created situation) in the student population [33]. Similar results were found in the study by Vladut and colleagues, where only students of medicine, dental medicine, and pharmacy were included. However, this research was also conducted during the beginning of the pandemic, that is, before the advent of the vaccine, and these are all possible factors that had an impact on the occurrence of stress in the student population.

Numerous studies have already indicated a cause-and-effect relationship between parafunctional habits and TMD. Therefore, it is of great importance to mention bruxism in addition to stress, considering that they are often connected and jointly affect the development and worsening of TMD symptoms [34]. In addition to stress, the results of the research by Bertazzo et al. suggest an association between bruxism and the consumption of alcohol, tobacco products, and coffee; however, there is still a need for stronger evidence based on research with greater methodological rigor [35].

Impaired mobility of the jaws, difficulties in mastication, and verbal and non-verbal communication are some of the symptoms of TMD [36,37]. The results of this research were in line with that. Namely, the subjects classified in the medium and severe TMD categories reported difficulties in performing the previously mentioned actions. What is interesting is that subjects classified in the medium TMD category according to the FAI reported slightly more significant disturbances in jaw mobility, while those with severe TMD reported notable difficulties with verbal and non-verbal communication.

It is important to emphasize that this research has certain limitations despite the large number of respondents. Considering that this study is of a cross-sectional design, it is not possible to draw any causal conclusions, and it is also not possible to exclude all confounding factors that could affect the interpretation of the results. A self-reported questionnaire assessment needs to be complemented by a clinical examination in order to form a valid diagnosis [38]. Hence, there are some symptoms that do not undoubtedly represent TMD but can act like TMD symptoms, like dental pain, otological pathologies, headaches from other causes, or some neuropathic conditions. However, FAI is a reliable and sensitive tool that, according to the literature, is well-used for epidemiological studies of TMD [39,40]. Furthermore, only students of the Faculty of Medicine of the University of Split were included in the research, and future research should also include faculties in the field of biomedicine from other universities. Then, given that the students were asked about their lifestyle habits, there is a possibility that they gave more socially acceptable answers or that the recollection of their own habits could be a confounding factor in this research.

## 5. Conclusions

In conclusion, the results of this research, as well as previous ones, confirm that in the population of medical, dental medicine, and pharmacy students, there is a certain proportion of students who have some form of TMD, and it is necessary to educate students during their formal education about the importance of timely diagnosis and the adoption of healthy oral and other lifestyle habits. Smoking, consumption of alcohol and coffee, i.e., caffeinated beverages, are risk factors for TMD that students and the general population can influence.

## Figures and Tables

**Table 1:** Comparison of sociodemographic data and habits between different biomedical studies (N = 676).

Parameter	Medicine N = 351	Dental Medicine N = 148	Pharmacy N = 177	*p*
Male gender (N, %)	88 (25.1)	25 (16.9)	28 (15.8)	0.019 *
Age (years)	22.4 ± 2.2	22.8 ± 2.2	22.0 ± 1.9	0.059 ^†^
Study year (N, %)				
1st year	29 (8.3)	18 (12.2)	32 (18.1)	<0.001 *
2nd year	91 (25.9)	13 (8.8)	27 (15.3)	
3rd year	78 (22.2)	27 (18.2)	55 (31.1)	
4th year	45 (12.8)	25 (16.9)	25 (14.1)	
5th year	55 (15.7)	20 (13.5)	38 (21.5)	
6th year	53 (15.1)	45 (20.4)	/	
Smoking (N, %)	65 (18.5)	36 (24.3)	42 (23.7)	0.216 *
Smoking period (years)	3.8 ± 3.3	6.0 ± 2.9	3.4 ± 3.4	0.001 ^†b^
Cigarettes/day	6.6 ± 7.6	10.2 ± 5.7	6.5 ± 7.7	0.026 ^†a^
Alcohol consumption (N, %)	269 (76.6)	124 (83.8)	147 (83.1)	0.090 *
Alcohol consumption frequency				
Several times a week	29 (10.8)	8 (6.5)	18 (12.2)	
Once a week	87 (32.3)	52 (41.9)	42 (28.6)	0.139 *
Once a month	153 (56.9)	64 (51.6)	87 (59.2)	
Coffee consumption (N, %)	275 (78.3)	108 (73.0)	131 (74.0)	0.334 *
Coffee consumption period (years)	6.0 ± 2.8	6.8 ± 3.4	6.0 ± 3.1	0.040 ^†c^
Coffee cups/day	1.6 ± 0.9	1.6 ± 0.8	1.7 ± 0.8	0.470 ^†^
ED consumption (N, %)	61 (17.4)	20 (13.5)	24 (13.6)	0.387 *
ED consumption frequency				
Several times a week	8 (13.1)	0 (0)	0 (0)	
Once a week	8 (13.1)	3 (15.0)	4 (16.7)	0.527 *
Once a month	45 (73.8)	17 (85.0)	18 (75.0)	

All data is presented as whole numbers (percentage) or mean ± standard deviation. Abbreviations: ED—energy drinks. * Chi-square. † One-way analysis of variance (ANOVA) with post hoc Scheffé test. ^a^ medicine vs. dental medicine; *p* < 0.05. ^b^ medicine vs. dental medicine; pharmacy vs. dental medicine; *p* < 0.05. ^c^ dental medicine vs. pharmacy; dental medicine vs. medicine; *p* < 0.05.

**Table 2:** Comparison of sociodemographic data and habits between different stages of TMD depending on the FAI score (N = 676).

Parameter	No TMD FAI 0–15 N = 245	Minor TMD FAI 20–45 N = 303	Medium TMD FAI 50–65 N = 95	Severe TMD FAI 70–100 N = 33	*p*
Male gender (N, %)	60 (24.5)	61 (20.1)	12 (12.6)	8 (24.2)	0.103 *
Age (years)	22.0 ± 2.0	22.5 ± 2.1	22.6 ± 2.1	22.1 ± 2.1	0.021 ^†^
Study					
Medicine	132 (53.9)	152 (50.2)	51 (53.7)	16 (48.5)	
Dental medicine	46 (18.8)	71 (23.4)	24 (25.3)	7 (21.2)	0.716 *
Pharmacy	67 (27.3)	80 (26.4)	20 (21.1)	10 (30.3)	
Smoking (N, %)	50 (20.4)	63 (20.8)	27 (28.4)	13 (39.4)	0.014 *
Smoking period	4.4 ± 2.2	5.5 ± 2.6	8.7 ± 2.7	8.7 ± 2.1	<0.001 ^†a^
Cigarettes/day	6.8 ± 7.6	7.8 ± 7.1	8.2 ± 7.9	7.9 ± 8.1	0.897 ^†^
Alcohol consumption (N, %)	196 (80.0)	245 (80.8)	76 (80.0)	23 (69.6)	0.207 *
Alcohol consumption frequency					
Several times a week	25 (12.8)	23 (9.4)	5 (6.6)	2 (8.7)	
Once a week	74 (37.8)	72 (29.4)	26 (34.2)	9 (39.1)	0.251 *
Once a month	97 (49.5)	150 (61.2)	45 (59.2)	12 (52.2)	
Coffee consumption (N, %)	189 (77.1)	231 (76.2)	69 (72.6)	25 (75.8)	0.945 *
Coffee consumption period	5.7 ± 3.0	6.2 ± 2.9	6.5 ± 3.1	6.5 ± 3.4	0.088 ^†^
Coffee cups/day	1.5 ± 0.7	1.7 ± 0.8	1.9 ± 1.2	2.4 ± 1.7	<0.001 ^†b^
ED consumption (N, %)	45 (18.3)	44 (14.5)	10 (10.5)	6 (18.1)	0.766 *
ED consumption frequency					
Several times a week	3 (6.7)	5 (11.4)	1 (10.0)	1 (16.7)	
Once a week	7 (15.6)	5 (11.4)	2 (20.0)	1 (16.7)	0.954 *
Once a month	35 (77.8)	34 (77.3)	7 (70.0)	4 (66.6)	

All data is presented as whole numbers (percentage) or mean ± standard deviation. Abbreviations: TMD—temporomandibular disorder; FAI—Fonseca anamnestic index; ED—energy drink. * Chi-square test. † One-way analysis of variance (ANOVA) with post hoc Scheffé test. ^a^ no TMD vs. medium TMD; no TMD vs. severe TMD; minor TMD vs. medium TMD; minor TMD vs. severe TMD; *p* < 0.05. ^b^ no TMD vs. medium TMD; no TMD vs. severe TMD; minor TMD vs. severe TMD; *p* < 0.05.

**Table 3:** Comparison of the JFLS score between TMD severities depending on the FAI score.

Parameter	No TMD FAI 0–15 N = 245	Minor TMD FAI 20–45 N = 303	Medium TMD FAI 50–65 N = 95	Severe TMD FAI 70–100 N = 33	*p*
Total JFLS score	1 (0–5)	5 (1–12)	10 (3–27)	13 (3–27)	<0.001 *
Mastication	0 (0–2)	2 (0–5)	5 (1–7)	6 (1–8)	<0.001 *
Mobility	0 (0–2)	0 (0–4)	3 (0–6)	5 (1–8)	<0.001 *
Verbal and non-verbal communication	0 (0–3)	1 (0–3)	4 (0–10)	5 (2–12)	<0.001 *

All data is presented as the median (interquartile range). Abbreviations: JFLS—jaw functional limitation scale; TMD—temporomandibular disorder; FAI—Fonseca anamnestic index. * Kruskal–Wallis test with post hoc Dunn’s test.

**Table 4:** Multivariable logistic regression of independent factors predicting no TMD status.

Parametar	OR	95% CI	*p*
Age	0.901	0.805 to 1.010	0.073
Male gender	0.835	0.462 to 1.511	0.552
Smoking	0.564	0.306 to 1.040	0.046
Alcohol	1.597	0.841 to 3.035	0.152
Coffee	0.548	0.310 to 0.969	0.038
PSQ score	0.062	0.014 to 0.282	<0.001
JFLS score	0.923	0.891 to 0.957	<0.001

Abbreviations: TMD—temporomandibular disorder; OR—odds ratio; CI—confidence interval; PSQ—perceived stress questionnaire; JFLS—jaw functional limitation scale.

## Data Availability

All raw data sets are available upon request to the corresponding author via e-mail.

## References

[1] Li D.T.S., Leung Y.Y. (2021). Temporomandibular Disorders: Current Concepts and Controversies in Diagnosis and Management. Diagnostics.

[2] Durham J., Steele J.G., Wassell R.W., Exley C. (2010). Living with Uncertainty: Temporomandibular Disorders. J. Dent. Res..

[3] Ryan J., Akhter R., Hassan N., Hilton G., Wickham J., Ibaragi S. (2019). Epidemiology of Temporomandibular Disorder in the General Population: A Systematic Review. Adv. Dent. Oral. Health.

[4] Valesan L.F., Da-Cas C.D., Reus J.C., Denardin A.C.S., Garanhani R.R., Bonotto D., Januzzi E., de Souza B.D.M. (2021). Prevalence of temporomandibular joint disorders: A systematic review and meta-analysis. Clin. Oral Investig..

[5] Jin L.J., Lamster I.B., Greenspan J.S., Pitts N.B., Scully C., Warnakulasuriya S. (2016). Global burden of oral diseases: Emerging concepts, management and interplay with systemic health. Oral Dis..

[6] Fillingim R.B., Ohrbach R., Greenspan J.D., Knott C., Diatchenko L., Dubner R., Bair E., Baraian C., Mack N., Slade G.D. (2013). Psychological factors associated with development of TMD: The OPPERA prospective cohort study. J. Pain.

[7] Tanaka E., Detamore M.S., Mercuri L.G. (2008). Degenerative disorders of the temporomandibular joint: Etiology, diagnosis, and treatment. J. Dent. Res..

[8] Cairns B., List T., Michelotti A., Ohrbach R., Svensson P. (2010). JOR-CORE recommendations on rehabilitation of temporomandibular disorders. J. Oral Rehabil..

[9] Ferrillo M., Migliario M., Marotta N., Fortunato F., Bindi M., Pezzotti F., Ammendolia A., Giudice A., Foglio Bonda P.L., de Sire A. (2023). Temporomandibular disorders and neck pain in primary headache patients: A retrospective machine learning study. Acta Odontol. Scand..

[10] Minervini G., Mariani P., Fiorillo L., Cervino G., Cicciù M., Laino L. (2022). Prevalence of temporomandibular disorders in people with multiple sclerosis: A systematic review and meta-analysis. Cranio.

[11] Murphy M.K., MacBarb R.-F., Wong M.-E., Athanasiou K.A. (2013). Temporomandibular disorders: A review of etiology, clinical management, and tissue engineering strategies. Int. J. Oral Maxillofac. Implants.

[12] Małgorzata P., Małgorzata K.M., Karolina C., Gala A. (2020). Diagnostic of Temporomandibular Disorders and Other Facial Pain Conditions-Narrative Review and Personal Experience. Medicina.

[13] González-González A.M., Herrero A.J. (2021). A systematic review of temporomandibular disorder diagnostic methods. Cranio.

[14] Schiffman E., Ohrbach R., Truelove E., Look J., Anderson G., Goulet J.-P., List T., Svensson P., Gonzalez Y., Lobbezoo F. (2014). Diagnostic Criteria for Temporomandibular Disorders (DC/TMD) for Clinical and Research Applications: Recommendations of the International RDC/TMD Consortium Network* and Orofacial Pain Special Interest Group. J. Oral Facial Pain Headache.

[15] Garrigós-Pedrón M., La Touche R., Navarro-Desentre P., Gracia-Naya M., Segura-Ortí E. (2018). Effects of a Physical Therapy Protocol in Patients with Chronic Migraine and Temporomandibular Disorders: A Randomized, Single-Blinded, Clinical Trial. J. Oral Facial Pain Headache.

[16] Madani A., Ahrari F., Fallahrastegar A., Daghestani N. (2020). A randomized clinical trial comparing the efficacy of low-level laser therapy (LLLT) and laser acupuncture therapy (LAT) in patients with temporomandibular disorders. Lasers Med. Sci..

[17] Marotta N., Ferrillo M., Demeco A., Drago Ferrante V., Inzitari M.T., Pellegrino R., Pino I., Russo I., de Sire A., Ammendolia A. (2022). Effects of Radial Extracorporeal Shock Wave Therapy in Reducing Pain in Patients with Temporomandibular Disorders: A Pilot Randomized Controlled Trial. Appl. Sci..

[18] Beecroft E.V., Durham J., Thomson P. (2013). Retrospective examination of the healthcare ‘journey’ of chronic orofacial pain patients referred to oral and maxillofacial surgery. Br. Dent. J..

[19] Ehde D.M., Dillworth T.M., Turner J.A. (2014). Cognitive-behavioral therapy for individuals with chronic pain: Efficacy, innovations, and directions for research. Am. Psychol..

[20] List T., Axelsson S. (2010). Management of TMD: Evidence from systematic reviews and meta-analyses. J. Oral Rehabil..

[21] Aggarwal V.R., Lovell K., Peters S., Javidi H., Joughin A., Goldthorpe J. (2011). Psychosocial interventions for the management of chronic orofacial pain. Cochrane Database Syst. Rev..

[22] Lei J., Yap A.U., Zhang M., Fu K.Y. (2021). Temporomandibular disorder subtypes, emotional distress, impaired sleep, and oral health-related quality of life in Asian patients. Community Dent. Oral Epidemiol..

[23] Sanders A.E., Maixner W., Nackley A.G., Diatchenko L., By K., Miller V.E., Slade G.D. (2012). Excess risk of temporomandibular disorder associated with cigarette smoking in young adults. J. Pain.

[24] de Leeuw R., Eisenlohr-Moul T., Bertrand P. (2013). The association of smoking status with sleep disturbance, psychological functioning, and pain severity in patients with temporomandibular disorders. J. Orofac. Pain.

[25] Miettinen O., Anttonen V., Patinen P., Päkkilä J., Tjäderhane L., Sipilä K. (2017). Prevalence of Temporomandibular Disorder Symptoms and Their Association with Alcohol and Smoking Habits. J. Oral Facial Pain Headache.

[26] Grgic J., Grgic I., Pickering C., Schoenfeld B.J., Bishop D.J., Pedisic Z. (2020). Wake up and smell the cofee: Cafeine supplementation and exercise performance—An umbrella review of 21 published metaanalyses. Br. J. Sports Med..

[27] Stasiak G., Maracci L.M., de Oliveira Chami V., Pereira D.D., Tomazoni F., Bernardon Silva T., Ferrazzo V.A., Marquezan M. (2020). TMD diagnosis: Sensitivity and specificity of the Fonseca Anamnestic Index. Cranio.

[28] Ohrbach R., Larsson P., List T. (2008). The jaw functional limitation scale: Development, reliability, and validity of 8-item and 20-item versions. J. Orofac. Pain.

[29] Levenstein S., Prantera C., Varvo V., Scribano M.L., Berto E., Luzi C., Andreoli A. (1993). Development of the Perceived Stress Questionnaire: A new tool for psychosomatic research. J. Psychosom. Res..

[30] Quadri M.F., Mahnashi A., Al Almutahhir A., Tubayqi H., Hakami A., Arishi M., Alamir A. (2015). Association of Awake Bruxism with Khat, Coffee, Tobacco, and Stress among Jazan University Students. Int. J. Dent..

[31] Benoliel R., Sela G., Teich S., Sharav Y. (2011). Painful temporomandibular disorders and headaches in 359 dental and medical students. Quintessence Int..

[32] Medeiros R.A., Vieira D.L., Silva E.V.F.D., Rezende L.V.M.L., Santos R.W.D., Tabata L.F. (2020). Prevalence of symptoms of temporomandibular disorders, oral behaviors, anxiety, and depression in Dentistry students during the period of social isolation due to COVID-19. J. Appl. Oral Sci..

[33] Vladutu D., Popescu S.M., Mercut R., Ionescu M., Scrieciu M., Glodeanu A.D., Stănuși A., Rîcă A.M., Mercuț V. (2022). Associations between Bruxism, Stress, and Manifestations of Temporomandibular Disorder in Young Students. Int. J. Environ. Res. Public Health.

[34] Chemelo V.D.S., Né Y.G.S., Frazão D.R., de Souza-Rodrigues R.D., Fagundes N.C.F., Magno M.B., da Silva C.M.T., Maia L.C., Lima R.R. (2020). Is There Association Between Stress and Bruxism? A Systematic Review and Meta-Analysis. Front. Neurol..

[35] Bertazzo-Silveira E., Kruger C.M., Porto De Toledo I., Porporatti A.L., Dick B., Flores-Mir C., De Luca Canto G. (2016). Association between sleep bruxism and alcohol, caffeine, tobacco, and drug abuse: A systematic review. J. Am. Dent. Assoc..

[36] List T., Jensen R.H. (2017). Temporomandibular disorders: Old ideas and new concepts. Cephalalgia.

[37] Gesch D., Bernhardt O., Mack F., John U., Kocher T., Alte D. (2005). Association of malocclusion and functional occlusion with subjective symptoms of TMD in adults: Results of the Study of Health in Pomerania (SHIP). Angle Orthod..

[38] Schnabl D., Sandbichler P., Neumaier M., Girstmair J., Barbieri F., Kapferer-Seebacher I., Steiner R., Laimer J., Grunert I. (2021). Dental Students’ Oral Health-Related Quality of Life and Temporomandibular Dysfunction-Self-Rating versus Clinical Assessment. Healthcare.

[39] Pires P.F., de Castro E.M., Pelai E.B., de Arruda A.B.C., Rodrigues-Bigaton D. (2018). Analysis of the accuracy and reliability of the Short-Form Fonseca Anamnestic Index in the diagnosis of myogenous temporomandibular disorder in women. Braz. J. Phys. Ther..

[40] Yarasca-Berrocal E., Huamani-Echaccaya J., Tolmos-Valdivia R., Tolmos-Regal L., López-Gurreonero C., Cervantes-Ganoza L.A., Cayo-Rojas C.F. (2022). Predictability and Accuracy of the Short-Form Fonseca Anamnestic Index in Relation to the Modified Helkimo Index for the Diagnosis of Temporomandibular Disorders: A Cross-Sectional Study. J. Int. Soc. Prev. Community Dent..

